# Fetal Oropharyngeal Teratoma: Prenatal Diagnosis and Imaging Characteristics

**DOI:** 10.7759/cureus.11329

**Published:** 2020-11-04

**Authors:** Ruchika Narayan, Md Shamim Ahmad, Amit Kumar

**Affiliations:** 1 Radiodiagnosis/Gastroenterology, Narayan Medical College and Hospital, Sasaram, IND; 2 Radiodiagnosis, Narayan Medical College and Hospital, Sasaram, IND

**Keywords:** oropharyngeal teratoma, fetal tumour

## Abstract

Fetal oropharyngeal teratoma (OPT) is an extremely rare disorder. This generally originates from the upper jaws that are connected to the hard palate. Pregnant women with fetal OPT usually present with oropharyngeal mass and polyhydramnios. Ultrasound can help in the pre-natal diagnosis of this condition, although magnetic resonance imaging (MRI) is useful for further characterization of the lesion. Because of severe obstruction to airways, OPTs are associated with high morbidity and mortality rates during peripartum period. We present here a case of fetal OPT with imaging characteristics with respect to the antenatal diagnosis.

## Introduction

Oropharyngeal teratoma (OPT) is an extremely rare congenital teratoma, occurring in 1 in 35,000-200,000 of live births [1,2]. It is also known as Epignathus. The bulk of the tumour cells tends to plug the oral cavity, often leading to a severe obstruction of the upper airways and high mortality rates during the neonatal period [3,4]. Earlier, OPT used to be identified very late, at times even after birth. Nevertheless, with the increase in knowledge and technological advancement in the ultrasonography (USG) enabling us a detailed characterisation of tumour, an early diagnosis of OPT during the antenatal period has become possible [2,4,5].

OPT usually presents with oropharyngeal mass associated with polyhydramnios [5]. The level of maternal serum alpha-fetoprotein (AFP) may be elevated. A pre-natal diagnosis of OPT can be made by USG. Magnetic resonance imaging ( MRI) is, however, helpful in detailed characterization of lesion, excluding central nervous system (CNS) involvement and determining the tracheal anatomy for airway safety [6]. Early diagnosis of OPT is of great value for obstetric and neonatal management. Because of severe obstruction to airways, OPT is generally associated with high mortality and morbidity rates during peripartum period [7,8]. This report describes the prenatal diagnosis and imaging characteristics of OPT on sonography and MRI.

## Case presentation

**Figure 1:**
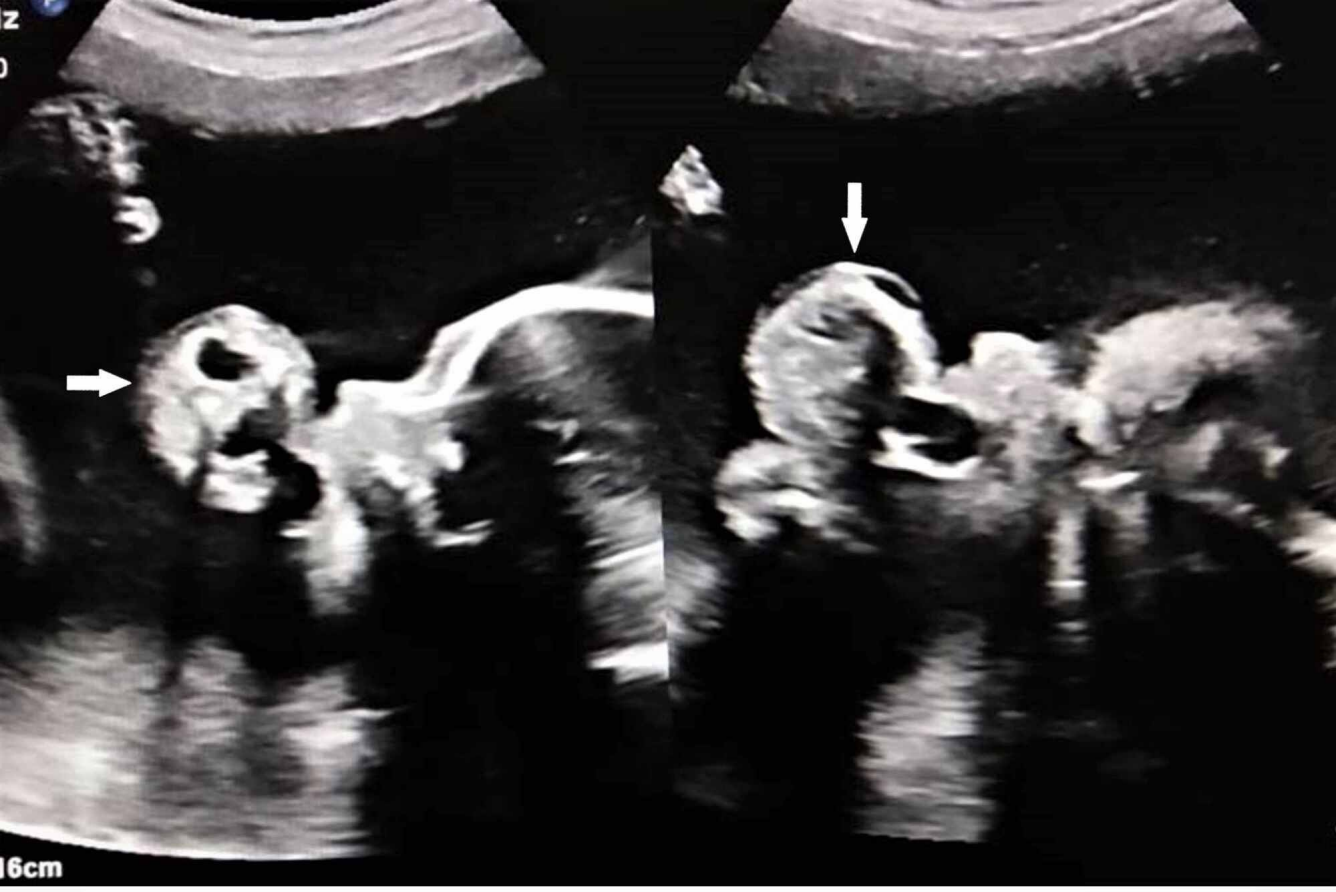
Ultrasound showing a pedunculated polypoidal mass protruding from the mouth of the fetus and freely floating in the amniotic cavity. The mass has mixed solid-cystic echotexture with areas of calcifications and minimal vascularity.

**Figure 2:**
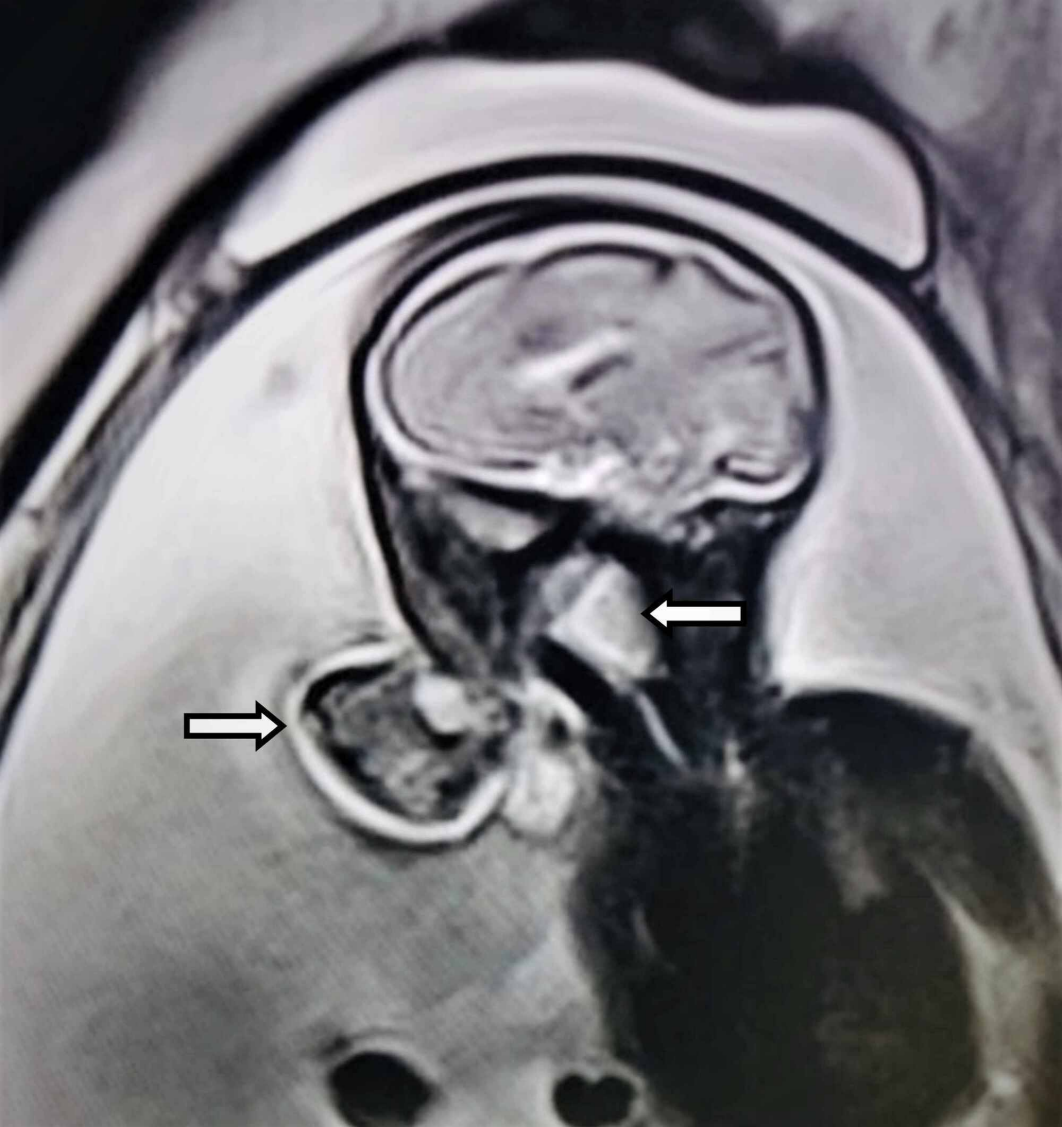
Sagittal MRI scan showing a mixed intensity mass with a stalk seen protruding through jaws into the amniotic fluid. The internal portion of mass emerging in relation to the hard palate and filling the oropharynx. There is no associated brain anomaly or intracranial mass invasion. MRI, magnetic resonance imaging.

**Figure 3:**
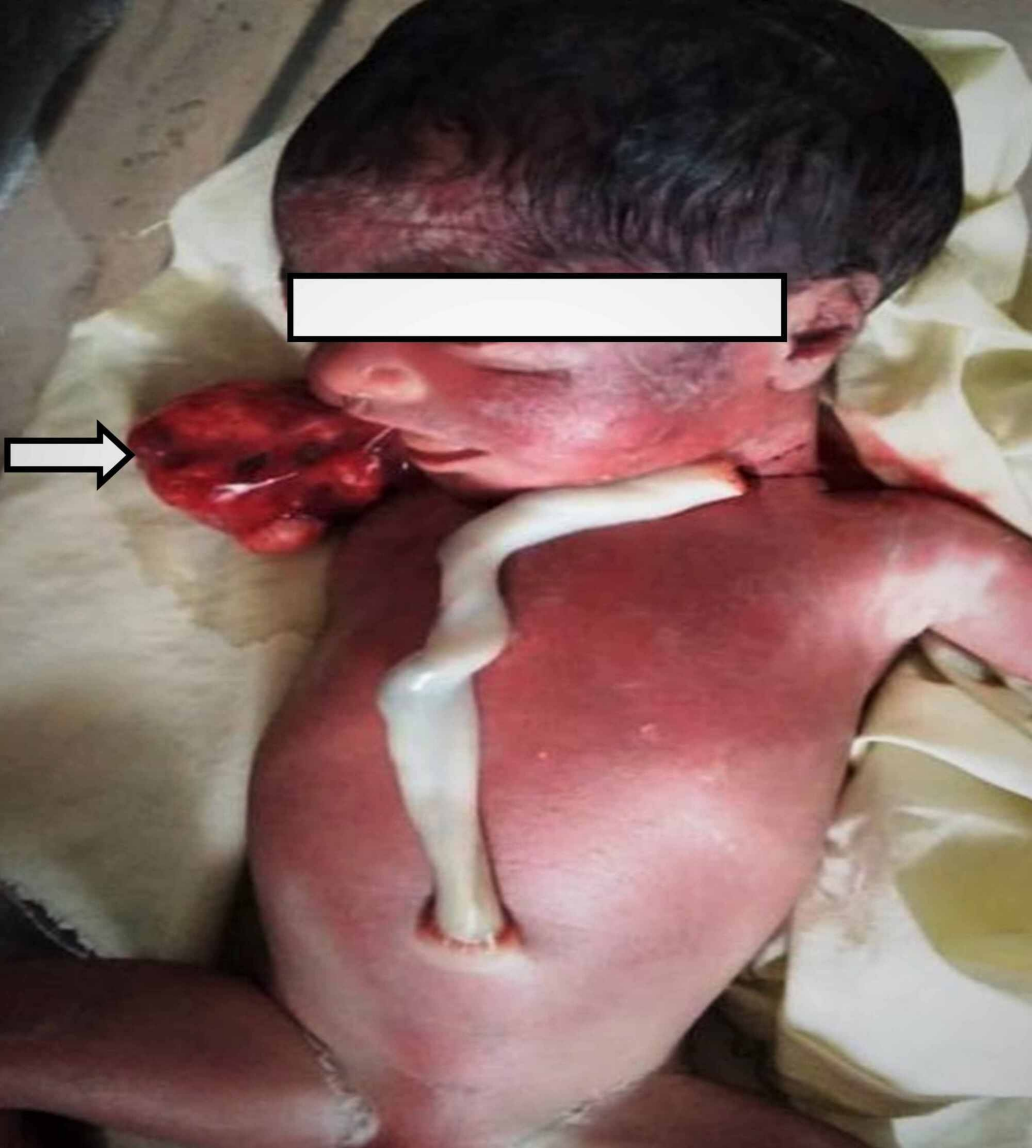
Fetus showing mass protruding from the oral cavity.

A 21-year-old pregnant gravid 1, para 1 women with no significant past history presented during 27th weeks of pregnancy with lower abdominal pain and increasingly abdominal girth. There was no family history of malformation or any hereditary disease. Clinically, there was suspicion of polyhydramnios. No biochemical screening test for aneuploidy (including AFP) had been performed during the second trimester. On USG, a pedunculated polypoidal mass was seen protruding from the mouth of the fetus and freely floating in the amniotic cavity (Figure 1). The mass measured 4.6 x 3.6 cm in size and had mixed solid-cystic echotexture. It also showed areas of calcifications and minimal vascularity. In addition, there was an associated polyhydramnios as indicated by amniotic fluid index (AFI >28.5). The cervical length was 3.5 cm. For further characterization of the tumor and to study the relationship of the tumor with surrounding structures, a fetal MRI was performed. On a sagittal MRI scan, a mixed intensity mass with a stalk was seen protruding through the jaws into the amniotic fluid. The internal portion of mass emerged with respect to the hard palate filling the significant part of the oropharynx. There was no associated CNS anomaly or intracranial invasion by mass (Figure 2). A day after imaging, the patient had a spontaneous delivery of a live foetus with mass protruding from the oral cavity (Figure 3). The mass was large, soft, and mobile with evidence of surface haemorrhage. No other fetal anomaly was identifiable. Before we could do anything, baby succumbed to death soon after the birth due to severe respiratory distress. Subsequently, histological analysis of mass confirmed the diagnosis of teratoma. The same woman now has a second pregnancy in the third trimester, and no fatal abnormalities have been found so far in her screening tests.

## Discussion

Teratomas are tumours consisting of all three layers of germ cells and account for around one-third of all neonatal tumours [9]. They can be located along the midline of the body from the head to the pelvis, but occur most often in the sacrococcygeal region. About 6%-10% of all teratomas are head and neck teratomas and are most commonly located in the cervical zone, with oropharynx being uncommon site (<1%) [10]. Therefore, OPT is an exceptionally rare form of fetal teratoma.

Classically, a case of OPT presents with oropharyngeal mass, polyhydramnios and elevated maternal serum AFP level [5,8]. An OPT appears as heterogeneous masses of solid and cystic components and are thought to occur due to trapping of mesoderm and endoderm with ectoderm during embryogenesis [9]. Due to the existence of both solid and cystic elements, OPT usually appears as heterogeneous mass on sonograms. The solid component consists of tissues of varying densities, such as liver, cartilage, bone and teeth, while neural, gastrointestinal, or respiratory lined cavities can form the cystic spaces [8]. 

Maternal serum AFP is a commonly used test during second trimester to assess a possible neural tube defect. An elevated level of AFP in maternal serum can lead to sonographic investigation and diagnosis of OPT [5,8]. AFP levels may, however, be within the normal range and are not specific to this condition. In our case, however, the patient had presented very late and the maternal serum AFP test had not been done during the second trimester, so the USG detected the condition first. This signifies that regular prenatal check-ups and screening tests are necessary in order to rule out a fatal defect earlier, a procedure that women living in rural and remote areas often ignore in the developing countries. Other conditions where maternal serum AFP levels may be elevated are pregnancies with foetal neural tube defects, autosomal trisomies, sacrococcygeal teratoma, and esophageal or anorectal atresia [11]. 

OPT, as in our case, frequently causes polyhydramnios due to the foetus' inability to swallow the amniotic fluid. Polyhydramnios is also a significant factor for prognosis, since it indirectly suggests the degree of airway obstruction and the extent of tumour invasion. Moreover, polyhydramnios is often associated with an increased risk of pre-term birth, pre-term premature membrane rupture and neonatal respiratory distress. Palatal clefting is also often caused by these tumours; however, there was no cleft in the palate of our case [12].

In general, USG is sufficient to diagnosis OPT in utero. Fetal MRI has complementary role and may be particularly helpful in determining the need for an EXIT procedure to be performed on a fetus [6]. By defining a relationship between the mass and airway structures, MRI is useful in ensuring airway patency. The prognosis oropharyngeal teratoma is generally very poor [7,8]. It is primarily dependent on the bulk of the lesion and the involvement of essential structures. Most deaths are due to obstruction of the airway. In a combined review of 33 neonates with oropharyngeal teratoma, twelve died in utero or shortly after birth [13]. In our case too, the neonate died shortly after birth because of severe respiratory distress before we could plan for any intervention. To the best of our knowledge, no cases of recurrence of OPT in subsequent pregnancies have been reported. Moreover, malignant transformation in OPT seldom occurs.

## Conclusions

Oropharyngeal teratomas are a rare fetal tumour that can be diagnosed with the use of ultrasounds in utero. For the further characterization of tumour and post-delivery management, such as ex-utero intrapartum treatment (EXIT) procedure, MRI may be helpful. However, the prognosis of this condition is extremely grave and death can occur immediately after birth due to severe respiratory distress. Nevertheless, parents can be reassured that they are not at increased risk of bearing another child with the same lesion.
